# Arginine: I. Interactions of Its Guanidinium Moiety with Branched Aliphatic Side Chains

**DOI:** 10.1021/acs.jpcb.5c02168

**Published:** 2025-07-09

**Authors:** Christopher M. Ng, Vivian Kui, Ruofan Li, Eric R. Kempson, Margaret Mandziuk

**Affiliations:** Department of Chemistry, 5894 New York University, New York, New York 10003, United States

## Abstract

The guanidine moiety of arginine side chains in proteins is often close to branched aliphatic side chains. We used the dispersion-corrected ωB97X-D density functional to calculate the interaction energy between the models of Arg and Leu side chains. We found that, in the lowest energy planar-like structures, the amino groups of methylguanidinium ion—our model of Arg side chains, and the methyl groups of 2-methylbutane—our model of leucine or isoleucine side chains, approach each other very closely. In these pairs, the methyl groups of Leu act as bases, donating electrons to the Arg side chains. The shortest distance between hydrogen atoms on different monomers is smaller than the sum of their van der Waals radii (2.2–2.4 Å). The shortest distance between a nitrogen atom of Arg and a carbon atom of Leu in these structures is around 3.4 Å. The stacked-like structures have a higher energy. The charge transfer in these pairs is an order of magnitude smaller. We inspected high-resolution files from the protein data bank (PDB), selected from the PISCES database, and found even closer approaches between Arg and Leu side chains in crystals. Stronger interactions between the side-chain models can be obtained by the second protonation of the guanidinium moiety of Arg. In our calculations for the doubly protonated dimer, the N···C distance decreases to about 3.1 Å, and the H···H distance becomes significantly lower than 2 Å. Our calculations, as well as the inspection of the PDB structures, pose the question of why Leu and Arg side chains approach each other so closely. Can Leu stabilize the doubly protonated guanidinium moiety of Arg, or does an exchange of protons occur between amino and methyl groups, as it does in dihydrogen-bonded complexes? Further experimental studies are needed to answer these questions.

## Graphical abstract

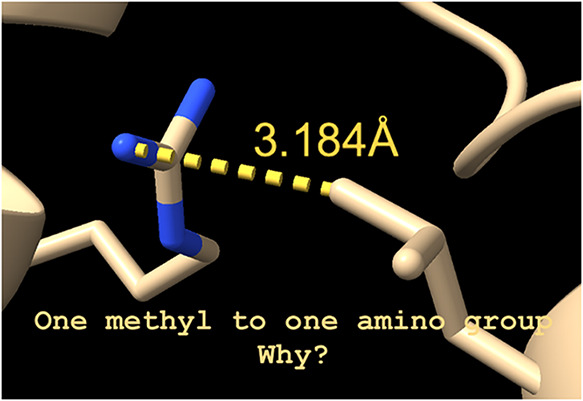

## Introduction

The amino acid (AA) arginine (Arg) is a key player in many biological processes. Its side chain is terminated with a guanidino (Gdn) group that can form multiple hydrogen bonds. Due to its very high p*K*_a_, the guanidine moiety is protonated even at internal positions in proteins.^1,2^ It forms hydrogen bonds predominantly in its plane, while surfaces below and above its plane are hydrophobic, often involving π–π contacts.^3^ Many charged side chains of arginine are located near the surfaces of proteins, where they form hydrogen bonds with water. However, arginine is also found in relatively hydrophobic interiors of proteins where it plays critical roles in many processes.^1^ For example, Arg residues of potassium channels, buried in membranes, are responsible for carrying a gating charge.^4^ Arginine-rich peptides are effective in penetrating cell membranes.^5^ With many common mutations at Arg sites, the p53 protein loses its tumor-suppressive function or even becomes an oncogene.^6^

It has been recognized that contacts between Arg and branched aliphatic residues, such as leucine (Leu), occur in proteins and are quite common. Singh and Thornton found in 1990 that in structures recorded in the Protein Data Bank (PDB) at that time,^7^ out of 227 Arg contacts with other side chains, 44 contacts were with leucine (Leu).^8^ Subsequently, Nandi et al. analyzed the atomic environments of Arg in proteins.^9^ They reported a wide range of distances between the Arg and methyl groups (between 2.8 and 5 Å). There was even a closer contact reported between 2.4 and 2.5 Å. However, in their work, there was no specific information about amino acids (AAs) to which these methyl groups belonged.^9^ More recently, Anderson et al. found that 9% of Arg close contacts within 5 Å are with Leu, the third highest number, after close contacts with negatively charged side chains of glutamic acid (Glu) and aspartic acid (Asp).^3^ This large number of contacts with Leu was attributed to the high frequency of Leu residues occurring in proteins.

The strength of Arg and Leu interactions between their side chains has been assumed to be small,^10^ although, to our knowledge, it has not been evaluated at a higher computational level. Arg is very often found in ion channels close to the branched aliphatic side chains of Leu, Ile, or valine (Val).^3,10^ Without some attraction between cationic and aliphatic side chains, the number of close contact pairs should be smaller.

Here, we report the results of structural optimization of a dimer formed between methylated guanidinium ions (mGdnH^+^) and 2-methylbutane (TMB), a model for interactions between Arg and Leu (or Ile) side chains. Methylated guanidinium ion has been used to model Arg side chains in the past.^11^ We used hydrogen atoms to cap the α-carbon of Leu (or Ile). We did not include oxygen or nitrogen atoms of peptide bond fragments in order to focus specifically on the interactions between the cationic group and purely hydrophobic species. This approach should be valid for side chains belonging to α-helices where C=O and N–H groups form hydrogen bonds along the helix axis and are not available to pair up with sticking out side chains.

Our calculations in a vacuum serve as a reference point for our further calculations in different environments. In part II of this paper,^12^ we show how the interaction energy of Leu–Arg close contact pairs changes upon Arg forming a salt bridge (SB) with an acidic residue (Ac). We also compare the changes in structure and energy in an environment with a higher dielectric constant of 4.9 (chloroform). In the packed environment of a crystal, cavities, or channels, water has a much lower dielectric constant.^13,14^ Our results presented in this paper, obtained for a vacuum, should be applicable to protein interiors, where the mean dielectric constant in the interior of proteins is low, estimated to be 3.23 ± 0.04.^15^

## Methods

### Computational Details

For optimization of counterpoise-corrected energies and structures, we employed the ωB97X-D density functional.^16^ The aug-cc-pVTZ basis set^17^ was used in all optimizations. Calculations were performed with the Gaussian16, version A03, suite of programs.^18^ Partial charges and the decomposition of the interaction energy were calculated with the Natural Bond Orbitals (NBO), Version 7.0.10 program,^19^ interfaced with Gaussian16, version C01.^20^ The input structures were created from the optimized structures of mGdnH^+^ and TMB in the GaussView 6 visualization platform.^21^

The ωB97X-D functional has been used with success in calculations of weakly bound complexes.^22–25^ Recently, Negi et al. used this functional to calculate energies of guanidinium ions interacting with individual amino acids.^25^ The amino and carboxy ends of their AAs were capped with methyl groups in order to simulate fragments of a polypeptide backbone. However, for AAs with aliphatic side chains, only the interactions with the backbone fragments were reported.^25^

### PDB Search and Analysis

In order to find close contacts pairs between Leu and Arg side chains, we selected a file on the PISCES server^26^ that contained a list of the PDB structures culled for the high resolution of 1 Å, or better, 15% sequence identity, and *R* factor of 0.2.^27^ The coordinates of these protein chains were downloaded from the PDB server^7^ on Feb 2, 2024. They were parsed with a Python script for close contacts, below 3.5 Å, between any pair consisting of a nitrogen atom of Arg and a carbon atom of Leu side chains. Molecular graphics and analyses were performed with UCSF Chimera.^28^

## Results and Discussion

The potential energy surface for the interaction between mGdnH^+^ and TMB is rather flat and contains multiple shallow minima. We do not report all of the local minima; however, we expect that the reported structures cover the energetic range of all possible interactions. In Table 1, we present the energies for the nine selected optimized structures. They are arranged in order of increasing strength of the interaction energy between monomers. Also, some geometric parameters of the dimers are gathered in Table 1. All nine optimized structures are shown in Figure S1. In Figure 1, we show two selected structures that represent different types of binding modes between mGdnH^+^ and TMB.

**Figure 1:**
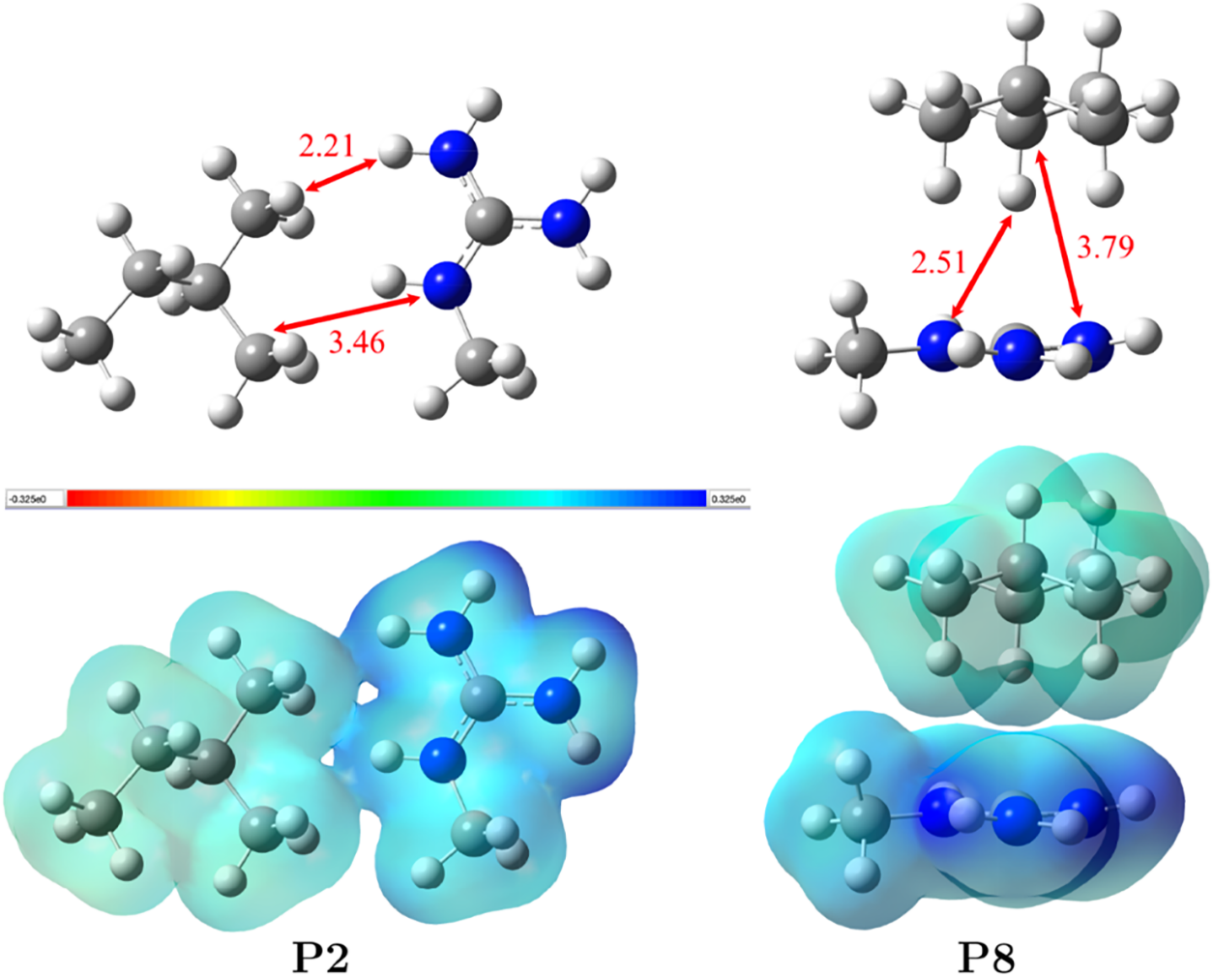
Structures of the two selected mGdnH^+^·TMB dimers. The shortest distances between hydrogen atoms belonging to different monomers are marked in the figure. Also, the shortest distances between carbons of TMB and nitrogens of mGdnH^+^ are indicated. Electrostatic potential (ESP) mapped on the iso-density surface at 0.007 e/bohr^3^ is shown below each structure. Refer to the text for further information.

**Table 1:** Energy Optimized at the ωB97X-D/aug-cc-pVTZ Level in Vacuum^a^

str. #	*E*_opt_ (E_h_)	*E*_ctps_ (E_h_)	Δ*E*_int_ (kcal/mol)	Δ*E*_bind_ (kcal/mol)	*r*(N·C)_min_ (Å)	*r*(H·H)_min_ (Å)
**P1**	–442.897719	–442.897307	–8.89	–8.40	3.424	2.063
**P2**	–442.897046	–442.896723	–8.31	–8.04	3.446	2.207
**P3**	–442.896175	–442.895858	–7.75	–7.49	3.479	2.137
**P4**	–442.895615	–442.895234	–7.42	–7.10	3.533	2.124
**P5**	–442.895019	–442.894706	–7.00	–6.77	3.429	2.041
**P6**	–442.893793	–442.893458	–6.49	–5.99	3.381	2.332
**P7**	–442.891955	–442.891642	–5.77	–4.85	3.538	2.564
**P8**	–442.892733	–442.892410	–5.55	–5.33	3.790	2.513
**P9**	–442.890673	–442.890457	–4.25	–4.10	3.300	2.874

a *E*_opt_, counterpoise-corrected energy; *E*_ctps_, interaction energy; Δ*E*_int_, binding energy; Δ*E*_bind_, calculated at the geometries optimized with the counterpoise correction. Also included are the shortest distances between the nitrogen of mGdnH^+^ and the carbon of TMB, as well as between the hydrogens for all structures. The Hessian of all structures is positive-definite.

The energy differences between some of the presented structures are small, and the energy order may change when using different calculation methods. However, some generalizations can be made. The structures with the lowest energies have hydrogens of two amino groups of the cation intercalated between the hydrogens of the hydrocarbon methyl groups in a planar-like fashion (see structures **P1**–**P3**). The interaction energy between mGdnH^+^ and TMB in these structures is in the range of 7–8 kcal/mol (we consider absolute values), which corresponds to 29–33 kJ/mol (kcal/mol = 4.184 kJ/mol). This is the range of medium strength hydrogen bonds; the water dimer is stabilized by about 5 kcal/mol. Typical binding energies of π–π^+^ interactions are in the range of 8–14 kcal/mol.^29^ In structure **P4**, mGdnH^+^ is inserted between the methyl groups of TMB, and it is perpendicular to the plane formed by the methyl and methylene groups. The energy of this structure is higher than that of the planar-like structures **P1**–**P3**. Structure **P5** has only one amino group of Arg guanidinium moiety intercalated between the methyl/methylene groups of Leu, and its energy is slightly higher than those of the **P1**–**P4** structures.

In the structures with a stacking-like arrangement, with TMB above the mGdnH^+^ plane (**P6**–**P9**), the magnitude of the interaction energy is lower. This is in the range of 4–6 kcal/mol. It is, however, slightly higher than the interaction between two aliphatic substances of similar size. Optimization of the energy of the 2-methylbutane dimer, (C_5_H_12_)_2_, at the same level of computations as used for the C_5_H_12_·GdnH^+^ dimer (ωB97X-D/aug-cc-pVTZ), yields an interaction energy of only 3.75 kcal/mol. The two optimized structures of the (C_5_H_12_)_2_ dimer are shown in Figure S2.

Our lower energy structures, **P1–P5**, reveal quite short H···H distances between mGdnH^+^ and TMB (see Table 1 and Figure S1). These distances are shorter than the sum of their van der Waals radii, 2.2–2.4 Å ^30,31^ (1 Å = 1 × 10^–10^ m). The interactions between monomers in these structures resemble those of dihydrogen bonding (DHB), where short H···H distances are observed.^30,31^ However, not all of the conditions of DHB, or H−σ bonding, specified by Grabowski,^31^ are satisfied for our dimers. Both hydrogens in close contact have positive partial charges; in DHB, one of the hydrogens should have a negative charge. Also, our dimers do not qualify as H−σ bonds with much stronger interaction energies.

In order to gain better insight into the interaction between guanidinium ion and the hydrocarbon, we found the components of the interaction energy with the NBO7 ^19^ program, interfaced with the Gaussian16, Version C01.^20^ The Natural Energy Decomposition Analysis (NEDA) of NBO7 considers three components to the interaction energy, Δ*E*_int_: (1) electrical (EL) component including the classical-like interaction between charges, bond dipoles, etc.; (2) steric exchange (EX) component representing Pauli exchange type repulsion between filled orbitals, or Lennnard-Jones-like repulsion between atoms; and (3) charge transfer (CT) component, which accounts for resonance-type delocalization between occupied orbitals on one fragment and unfilled orbitals on the other fragment. The components of the energy interactions between mGdnH^+^ and TMB are presented in Table 2.

**Table 2:** Optimized Energy, *E*_opt_, Interaction Energies Decomposed into the CT, EX, and EL Components vs Net Interaction Energy, Δ*E*_int_, and Charge (*q*)

str. #	*E*_opt_ (E_h_)	CT (kcal/mol)	EX (kcal/mol)	EL (kcal/mol)	Δ*E*_int_ (kcal/mol)	*q* (e)
**P1**	–442.897719	–10.797	3334.827	–3332.922	–8.892	0.01439
**P2**	–442.897046	–8.581	2617.159	–2616.884	–8.306	0.01229
**P3**	–442.896175	–8.147	2212.206	–2211.813	–7.754	0.01251
**P4**	–442.895615	–9.532	2798.409	–2796.293	–7.416	0.01371
**P5**	–442.895019	–8.396	2538.641	–2537.245	–6.999	0.01163
**P6**	–442.893793	–7.753	3831.487	–3830.225	–6.491	0.00360
**P7**	–442.891955	–6.497	3843.242	–3842.510	–5.766	–0.00007
**P8**	–442.892733	–6.284	3468.692	–3467.960	–5.552	–0.00089
**P9**	–442.890673	–3.191	2268.941	–2269.996	–4.246	0.00002

For the geometries optimized with the counterpoise correction, the EX and EL components add up to positive values. Without the CT contribution, the interaction between mGdnH^+^ and TMB would be repulsive for each structure, except for **P9**. The magnitude of the interaction energy is slightly lower than that of the CT component. The planar-like structures have the CT component with the largest magnitude; however, the stacked-like structures are stabilized by CT as well, despite the nearly zero net transferred charge.

NBO7 also identifies pairs of orbitals that provide the largest contribution to the charge transfer energy.^19^ The close H···H contacts between hydrogens of amino groups and hydrogens of methyl groups facilitate the interaction between their σ and σ* NBOs by bringing the bonds closer together and increasing their overlap.

In Figure 1, we show the electrostatic potential (ESP) mapped on the electron density isosurface for two representative structures: **P2** with lower energy and **P8** with higher energy. Each isosurface is drawn at a 0.007 e/bohr^3^ value, much higher than the usual default density value of 0.0004 e/bohr^3^ (bohr = 52.9 pm), which approximates space-filling-like electron clouds. The electrostatic potential in the figures ranged between −0.325 and 0.325 au ESP (1 au ESP = 27.211366 V). In Figure S1, these surfaces are shown for all of the reported structures.

The surfaces drawn at 0.007 e/bohr^3^ (e = 1.602177 × 10^–19^ C) show “funnel”-like electron density between monomers in low-energy structures. These “funnels” are due to CT interactions between NBOs of N–H bonds of mGdnH^+^ and NBOs of C–H bonds of TMB. The orbitals contributing the most to the CT energy are those with the shortest H···H distances (compare the location of the funnels with the short H–H distances in Figure S1). For example, for structure **P1**, the dominant interaction of 1.33 kcal/mol is between orbitals involving hydrogens separated by 2.06 Å. This is followed by interaction of 0.98 kcal/mol between bonds with a hydrogen separation of 2.15 Å. In low-energy structures, the dominant CT interactions are from mGdnH^+^ bonds to TMB bonds, resulting in a positive charge on TMB.

For lower energy stacking-like structures, interactions between NBOs on different monomers have much smaller magnitudes; however, there are more interacting pairs. Also, CT interactions of similar magnitude, occurring in the opposite direction, from TMB to mGdnH^+^ are present. The charge of the monomers is barely changed.

Note that structures **P1** and **P6** are better models for complexes between the side chains of Ile and Arg. Both of these structures have lower energies than the structures representing Leu·Arg interactions. Although less frequent than Leu in proteins,^3^ Ile may play an important role in interacting with cationic residues as well.

Attractive interaction between an aliphatic substance and mGdnH^+^ ion explains the presence of Arg side chains extending from membrane proteins into the lipid membrane environment in the voltage-sensing domain of the potassium channel (PDB ID 2R9R).^4,32,33^ The interaction between neutral methyl guanidine and TMB is also attractive. However, the interaction energy of a neutral complex is lower, around 4.5 kcal/mol (absolute value). The two structures of the neutral complex and their energies are shown in Figure S3. We did not obtain a planar-like structure for the neutral pair. Also, TMB acquired a negative charge in the neutral complex (Figure S3). Our structure, **P9**, is the only structure that has a lower interaction energy than neutral structures. These results indicate that it is more likely that Arg side chains inside a membrane are charged, confirming the work of Harms et al.^1^

In proteins, methyl groups were found to form attractive noncovalent interactions with carbonyl groups.^34,35^ However, methyl groups often behave as Lewis bases as well.^36^ In our calculations, methyl groups in low-energy structures act as electron donors.

The dynamics of Arg and Leu side chains in a close approach may not be that simple to elucidate computationally. Electronic structure optimization yields only static, rigid structures. It is known that guanidinium side chains of arginines rotate in proteins, and *N* ^η^ atoms are indistinguishable.^37^ The amino groups exchange hydrogens with the surroundings.^38,39^ Methyl groups display rotational tunneling at low temperatures.^40^ In proteins, they rotate fast at ambient conditions.^41^ When the side chains of Arg and Leu approach each other, is their motion restrained, or does it become more complex? Scrambling of dihydrogen-bonded protons was observed in dihydrogen-bonded complexes involving transition metals.^30^

We will show later that the distances between the nitrogen atoms of Arg and the carbon atoms of Leu in the close contact pairs, selected from the PDB, are shorter than the distances obtained in our calculations. Based on our results indicating that charge transfer strengthens interactions in the dimer, we postulated that the interactions between Leu and a doubly protonated Arg side chain should be stronger. Olah and White detected GdnH_2_^2+^ in “Magic Acid” in a ^1^H NMR experiment at −80 °C.^42^ The ion was also studied computationally.^43^ More recently, the ion was detected in crystals with superacids.^44^

Would Leu contribute to the stabilization of an extra proton on a guanidinium ion? Alkanes can be protonated, forming pentacoordinated, nonclassical carbonium ions, of which CH_5_^+^ is considered the parent.^45,46^ The CH_5_^+^ ion, discovered in mass spectroscopic experiments, displays very complex dynamics even at low temperatures.^47–50^ A close approach of a methyl group and a doubly protonated guanidine moiety may display interesting dynamics as well. Given that methyl groups have been used in NMR as probes for biomolecular interactions,^41^ they may provide insight into the interaction of Arg·Leu side chains.

We optimized a few structures of TMB and mGuaH_2_^2+^ dimers. The results are shown in Table 3. The energy of these dimers is lower, and the interaction energy (absolute value) is increased by a factor of about 3 relative to the interaction energy of the singly protonated dimers. Also, charge transfer is increased by a factor of about 5. The shortest intermolecular H···H distances in the doubly protonated dimers are well below 2 Å. The structures of these dimers are shown in Figure S4.

**Table 3:** Energy, *E* [E_h_], Interaction Energy, Δ*E*_int_ [kcal/mol], Binding Energy, Δ*E*_bind_ [kcal/mol], of Diprotonated TMB and mGuaH_2_^2+^ Dimers^a^

	ωB97X-D/aug-cc-pVTZ					
str. #	*E*	Δ*E*_int_	Δ*E*_bind_	TMB charge (au)	*r*(N···C)_min_ (Å)	*r*(H···H)_min_ (Å)
**D1**	–443.050913	–26.31	–21.66	0.07249	3.114	1.486
**D2**	–443.053041	–25.04	–23.00	0.06211	3.096	1.658
**D3**	–443.055841	–25.03	–24.75	0.04797	3.182	1.805
**D4**	–443.052545	–24.77	–22.69	0.07049	3.134	1.728
**D5**	–443.053249	–24.53	–23.13	0.06377	3.165	1.513
**D6**	–443.046916	–23.89	–19.15	0.06747	3.105	1.440
**D7**	–443.048140	–23.74	–19.92	0.05381	3.074	1.731
**D8**	–443.046810	–20.78	–19.09	0.05280	3.409	1.732

a All eigenvalues of the Hessian are positive. Also, the charge of TMB, and the shortest distances N···C and H···H between monomers are shown.

In order to determine the stability of a doubly protonated Leu–Arg side-chain complex in proteins, interactions with other interacting groups must be taken into account. However, our calculations indicate that the involvement of Leu–Arg side chains in transient protonation or proton transfer should be further considered, especially in hydrophobic membrane proteins.

How do our structures, optimized in vacuum, compare with those observed in proteins? In our calculated low-energy monoprotonated structures, distances between the carbon of Leu and the nitrogen of Arg are around 3.4 Å. In doubly protonated structures, this distance is shorter, around 3.1 Å. Such short distances between the nitrogen atoms of Arg side chains and the methyl carbons in proteins were reported in the early work of Nandi et al.^9^ However, the authors did not specify which AAs were involved in these short contacts. Currently, several proteins analyzed in this early work are no longer available in the PDB. In the more current review of Leu–Arg close contact pairs, Anderson et al. used a larger cutoff of distances between C···N atoms (5 Å) and presented relative positions of the two side chains only statistically.^3^

We visually inspected some structures with close contacts between the Leu and Arg side chains. All of the high-resolution files analyzed by us come from proteins crystallized from aqueous solutions. The majority of close contact pairs are on the surface of proteins or in cavities exposed to water. In the set of 270 protein chains, we found 89 pairs of Arg·Leu that fulfill the requirement of having the shortest C···N distance below 3.5 Å. We counted pairs formed in a biological unit, including pairs formed between residues at alternative locations. However, when Arg formed close contacts with two neighboring Leu residues, or when Leu formed close contacts with two neighboring Arg residues, we included such a triplet only once.

Approximately 60% of close contacts between Arg and Leu residues occur when the guanidinium moiety of the pair forms a salt bridge (SB) with a carboxylate group of Asp, Glu residue, or an OXT terminal group. In this article, we focus on Arg–Leu close contact pairs without SB interactions. The complexes containing Leu interacting with an SB are described and compared with optimized structures in part II of this paper.^12^ We used a criterion that SB was present when the distance between any of the nitrogens of the guanidinium moiety and any of the oxygens of the carboxylate group of Asp, Glu, or OXT was less than 3.5 Å. When the guanidinium moiety formed a salt bridge with an acidic residue of another chain not included in a biological unit, the pair was treated as being involved in an SB.

We found 38 pairs with close contact (<3.5 Å) between Arg and Leu side chains in which the guanidinium moiety of Arg did not form an SB. All the identified pairs are listed in Table S1. Our search yielded contacts that were even closer to those obtained in the calculations for the monoprotonated pairs. In one of the Arg·Leu pairs, the shortest N···C distance was below 3.0 Å. In two pairs, the shortest N···C distance was between 3.0 and 3.1 Å. We found one pair with the shortest distance between 3.1 and 3.2 Å. In six pairs, the shortest distance was in the range of 3.2–3.3 Å. In seven pairs, it was between 3.3 and 3.4 Å; in 20 pairs, it was between 3.4 and 3.5 Å.

In the majority of the close contact pairs, Leu side chains approach the guanidinium side chains from various angles with only one methyl group. Interestingly, a structure with a single methyl group perpendicular to the guanidinium plane, which in our calculations had the highest energy, was found as well. Three examples of structures with one methyl group approaching one amino group are presented in Figure S5a. We found only four structures with stack-like arrangements. Three of these are shown in Figure S5b. In many instances, there is an aromatic ring in the vicinity of the Leu·Arg close contact pair (Figure S5c).

Among the 38 close contact pairs, we found two pairs of Leu and guanidinium moieties in a planar-like orientation (PDB ID: 1MN8 and 5SBQ). These pairs are shown in Figure 2a,2b. They differ from our optimized structures by having a close contact between just one amino and one methyl group. (The structure with one close contact is a transition state in our calculation (see Figure S6).) In the Arg33·Leu13 pair from chain D of the PDB ID 1MN8 file, the other amino group forms a hydrogen bond with a water molecule that prevents the formation of the second contact. It is intriguing to see the very short distance between the Val36 and Arg33·Leu13 close contact pair in the PDB ID 1MN8 file. Val36 is above the pair, approaching Arg33 at a distance of 2.839 Å, which is much shorter than the 3.384 Å distance between the nitrogen of Arg33 and the carbon of Leu13.

**Figure 2:**
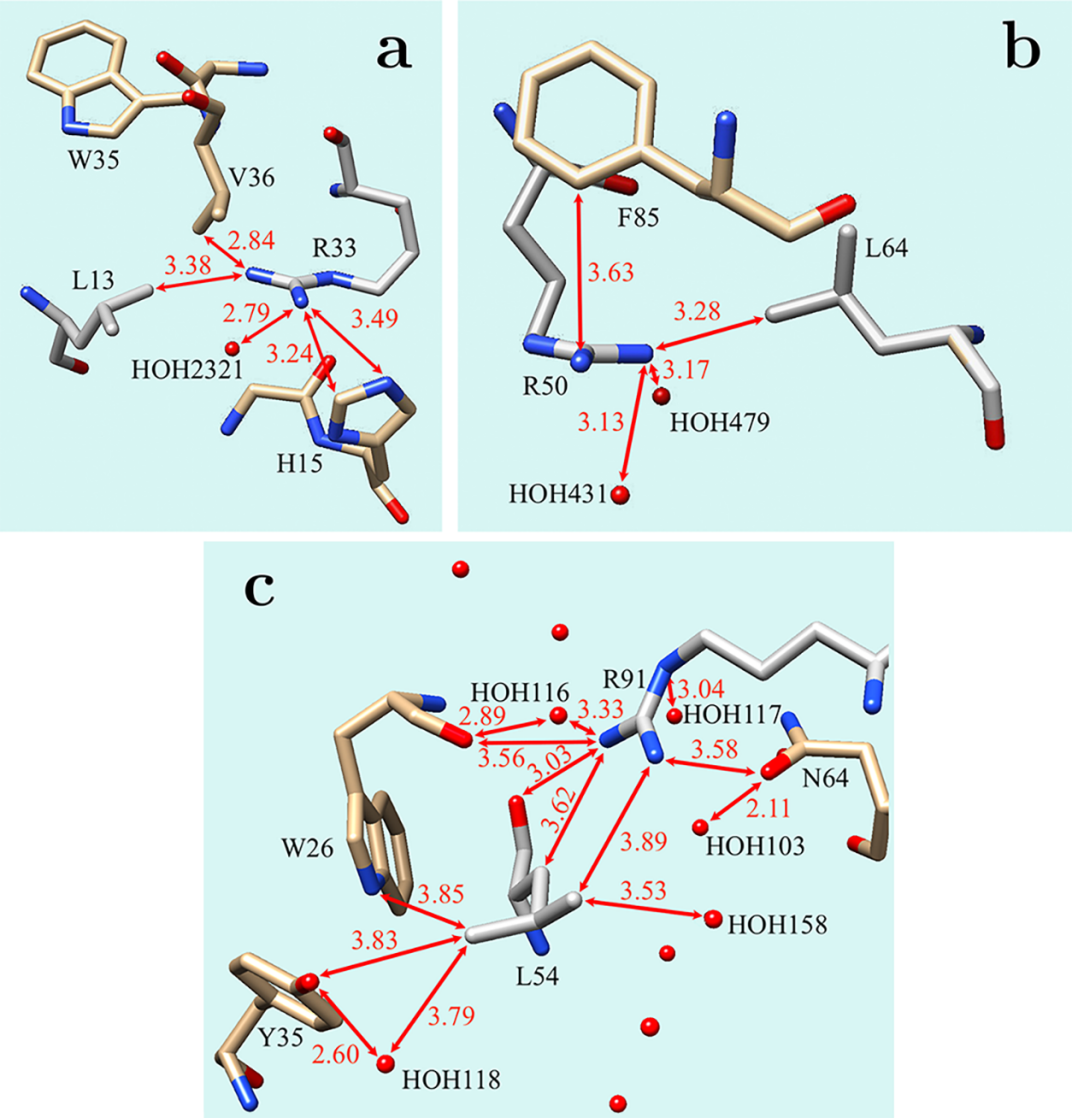
Close contact structures with a planar-like orientation of the guanidinium moiety of Arg and Leu, extracted from the PDB. (a) Arg33·Leu13 pair from chain D of the PDB ID 1MN8 file, (b) Arg50·Leu64 pair from chain A of the PDB ID 5SBQ file (Leu in alternative location A), and (c) Arg91·Leu54 pair from chains 2 and 4, or chains 5 and 8, of the unit cell in the PDB ID 7JGT file.

In the Arg50·Leu64 (loc.A) pair from chain A of the PDB ID 5SBQ file, the amino group in close contact with the methyl group of Leu additionally forms two hydrogen bonds with water molecules. The other two nitrogen atoms of the Arg side chain are less than 4 Å from the phenylalanine ring. A common feature of both pairs is the presence of an aromatic residue in their vicinity.

Although we did not actively search for Arg·Leu pairs formed in a crystal between chains of different biological units, while inspecting the structure of fibronectin type-III domain-containing protein (PDB ID 7JGT), we found an Arg·Leu pair in a planar-like orientation. This pair is shown in Figure 2c. Arg and Leu residues are present on the surfaces of different biological units; however, they are in the same crystal unit cell. In this pair, both amino groups of Arg interact with methyl groups of Leu, although the C···N distances are longer than our cutoff value of 3.5 Å. The shorter distance, at 3.618 Å, is on the side of the Arg residue facing the protein, while the longer distance, at 3.893 Å, is on the side exposed to water. As shown in Figure 2c, aromatic side chains (Trp26 and Tyr35) are also present in the vicinity of Leu54. Surprisingly, there are many water molecules around the pair that did not displace Leu from approaching the guanidinium moiety in a planar-like orientation. Charge-enhanced hydrogen bonding to water molecules, or to oxygen atoms in polar side chains, is significantly stronger than hydrogen bonding between two neutral species.^51^ These “charge-enhanced” hydrogen bonds, formed in the plane of the guanidinium ion, are expected to be stronger than the interaction with aliphatic Leu. Consequently, Leu is expected to be displaced to a position above or below the guanidinium planar surface. At the moment, we have no explanation for why a planar-like contact between Arg and Leu is stable in the presence of water.

Our calculations in vacuum should be valid for an environment of protein interiors with a low dielectric constant. However, the crystallographically determined structures of the close contact pairs on the surface of proteins resemble those obtained in our computations (see, for example, Figures 2c or S5). The first hydration layer of proteins is quite heterogeneous, and the dielectric constant varies from one residue to another. The value of the dielectric constant for bulk water at room temperature is around 80. Mondal et al. found a huge variation in the dielectric constant around the surface of a few proteins, lowering the average to around 45.^52^ Depending on the location/residue, the relaxation time of water at the protein surface varies by a factor of 10. In bulk water, only a 10% variation of the relaxation time is observed.^52^ An anomalously low dielectric constant of nanoconfined water was obtained in simulations^14^ and observed experimentally.^13^ In crystals, there are cavities between protein globules, where the dielectric constant may be substantially lowered.

How well does the structure of a protein surface in a crystal represent the structure of this surface in water or the environment of other proteins, condensates, etc.? There have been conflicting reports about the protonation state of arginine in the photoactive yellow protein in NMR and neutron diffraction studies. In the NMR studies, Yoshimura et al. detected six protons on the guanidinium moiety of R52.^53^ This was contrary to the earlier neutron diffraction work, where only five protons were found.^54^ Based on the work of Nadal-Ferret et al.,^55^ Yoshimura et al. suggested that this discrepancy arises from intrinsic differences in the structures of the protein in a crystal and in a solution.^53^

The environment of the Arg·Leu pair has an effect on the strength of their interactions. However, it will usually lower, rather than increase, their interaction strength. An example of the lowering interaction energy between Leu and Arg is presented in the accompanying paper II.^12^ In this case, Arg forms an SB with an acidic residue.

Interestingly, Leu approaches the amino group closely, even when this amino group is involved in a hydrogen bond with water or the oxygens of carbonyl groups (see Figures 2c or S5). Is there a special interaction between a methyl group and an amino group? Does the character of the interaction between Arg and Leu side chains change when the distance between these side chains increases beyond 3.5 Å? Do aromatic residues play a role in enhancing the interactions between Arg and Leu side chains? Are the nonpolar surroundings of Arg side chains on protein surfaces preparing the side chains for the formation of strong salt bridges in protein complexes?^12^ Further investigations are needed to delve deeper into the dynamics of Arg·Leu close contact pairs and to elucidate their role in proteins. It would also be interesting to ascertain the differences in Arg interactions with other branched aliphatic side chains, Ile and Val, or with other residues containing methyl groups.

## Conclusions

We used the dispersion-corrected ωB97X-D density functional to calculate the interaction energy between the models of Arg (methylguanidinium ion) and Leu (2-methylbutane) side chains. The optimized, lowest energy structures have very short distances between hydrogen atoms of Leu and hydrogen atoms of Arg amino groups, smaller than the sum of their van der Waals radii (2.2–2.4 Å). These close H···H contacts resemble dihydrogen bonding, with methyl groups donating electrons to the guanidinium ion. The low-energy structures have planar-like arrangements of guanidinium ion and Leu, with amino groups of Arg intercalated between the methyl/methylene groups of Leu. The shortest distance between the nitrogen of Arg and the carbon of Leu in these structures is around 3.4 Å. Stacking-like structures have higher energies and larger distances between their heavy atoms.

Our optimized structures resemble those found in the PDB, although the inspected PDB structures yielded even closer approaches between Arg and Leu side chains. Stronger interactions between the side-chain models were obtained by the second protonation of the guanidinium moiety of Arg. In our calculations of the doubly protonated dimer, the N···C distance decreased to about 3.1 Å, and the H···H distance became significantly lower than 2 Å. Our calculations and the inspection of the PDB structures pose the question of why Leu and Arg side chains approach each other so closely. Further experimental studies are needed to determine if Leu can stabilize the doubly protonated guanidinium moiety of Arg, or if an exchange of protons occurs between amino and methyl groups, as it does in dihydrogen-bonded complexes.

## Supplementary Material



## Notes

Optimized structures of mGdnH^+^·TMB dimers (Figure S1); energies and structures of two TMB dimers (Figure S2); energies and structures of two neutral mGdn·TMB dimers (Figure S3); optimized structures of mGdnH_2_^2+^·TMB dimers (Figure S4); examples from PDB (Figure S5); structure of TS with one methyl group approaching the amino group (Figure S6); identified Arg–Leu pairs in close contact (the shortest distance between the C atom of Leu and the N atom of the Arg side chain is less than 3.5 Å) (Table S1) (PDF)

## †

The Fu Foundation School of Engineering, Columbia University, New York, New York 10027, United States
