# Supplementary material for: Arginine: I. Interactions of Its Guanidinium Moiety with Branched Aliphatic Side Chains
Source: J Phys Chem B. 2025 Jul 9;129(29):7421–9. doi: 10.1021/acs.jpcb.5c02168 (PMC12302063; doi:10.1021/acs.jpcb.5c02168)
Supplement: Supplementary file 1 [file jp5c02168_si_001.pdf]

Supporting Information

Arginine: I. Interactions of its Guanidinium Moiety with Branched Aliphatic Side Chains

Christopher M. Ng, Vivian Kui, Ruofan Li, Eric R. Kempson, Margaret Mandziuk
Department of Chemistry, New York University, New York, USA

Figure S1: Optimized structures of methylated guanidinium ion and 2-methylbutane. The shortest distances between two hydrogen atoms on different monomers is marked (Å). Also, the electrostatic potential on the iso-density surface at 0.007 e/bohr^3 is shown.

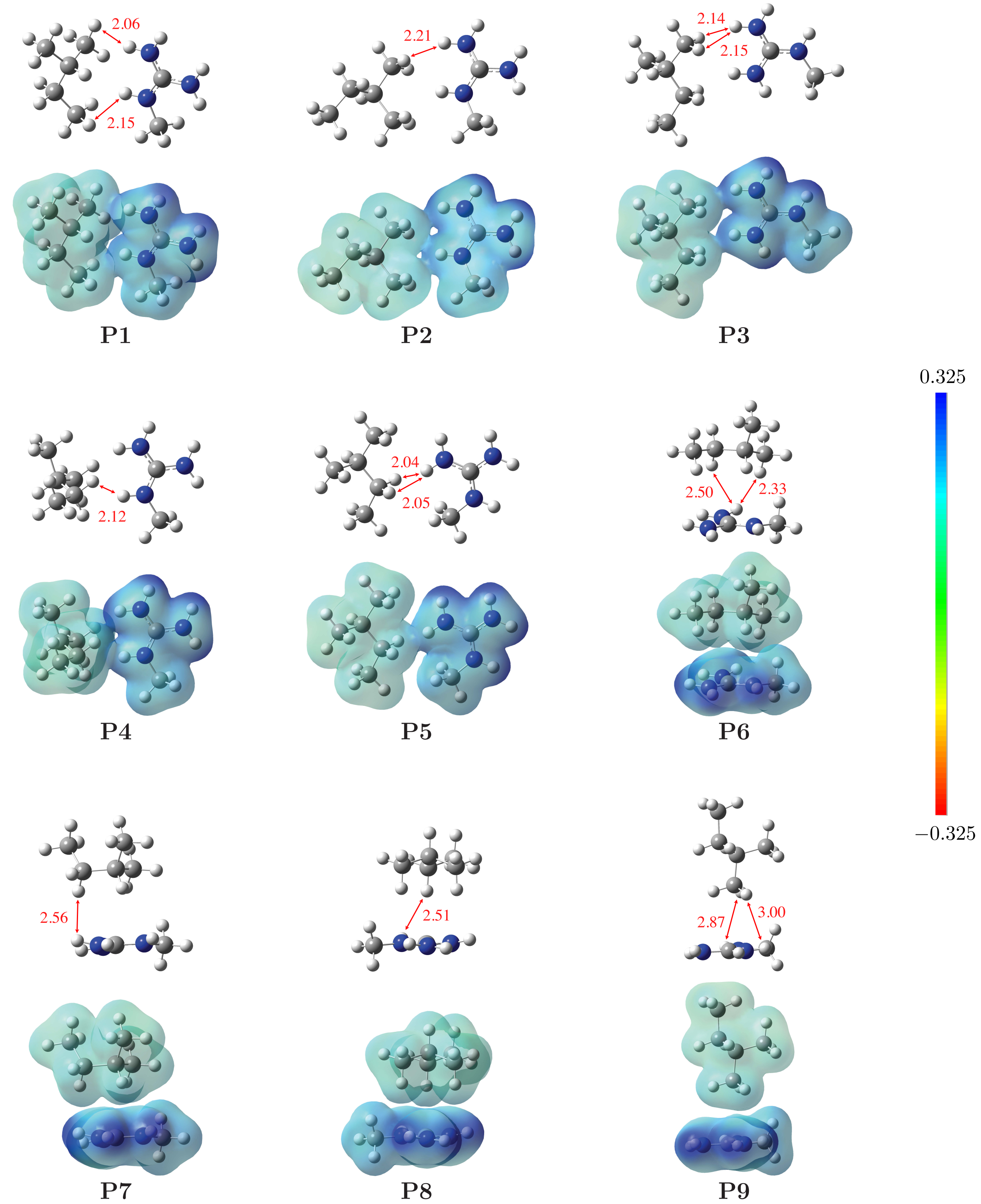

Figure S2: Optimized, counterpoise corrected interaction energies and structures of 2-methylbutane dimers. Dimers were optimized at ωB97X-D/aug-cc-pVTZ level. The lowest energy dimer is shown first. The second dimer was selected to show the range of interaction energy. Minimum distance between hydrogen atoms on different monomers is marked (in Å).

$(C_5H_{12})_2$

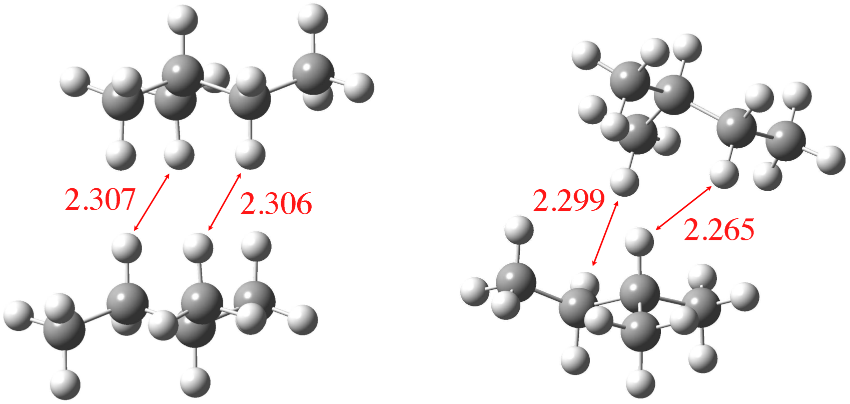

Interaction Energy (kcal/mol) (absolute value)	3.75	2.86
Energy (E_h)	−395.570624	−395.569336

Figure S3: Low energy, neutral methyl guanidine and 2-methylbutane (mGdn·TMB) structures have staking-like structures. The planar-like structures are not stable — the mGdn·TMB dimer, starting with a planar structure, lowers its energy by increasing surface contacts. The two optimized structures of neutral mGdn·TMB are presented below. The interaction energy, the counterpoise corrected total energy, and the NBO calculated charge of TMB in the dimer are given for each structure.

mGdn·TMB

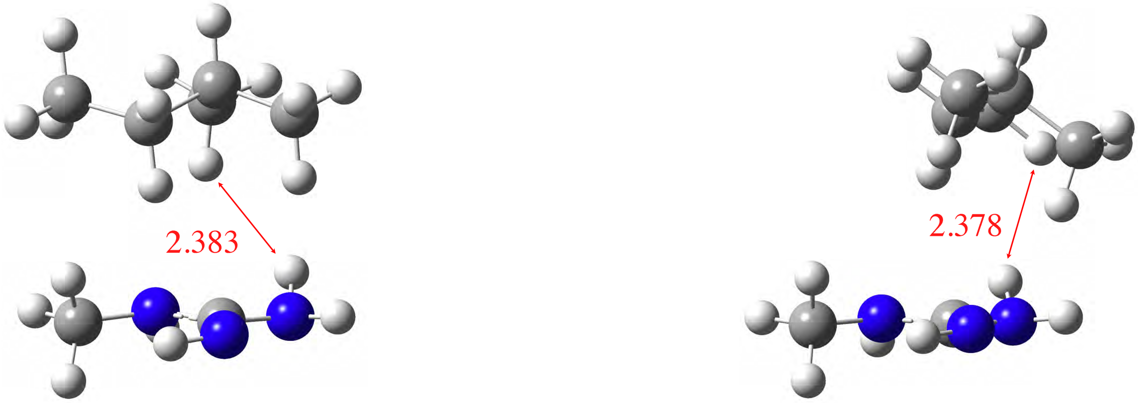

4.54	Interaction Energy (kcal/mol) (absolute value)	4.45
−442.489252	Energy (E_h)	−442.489283
−0.00488	Charge of TMB (e)	−0.00443

Figure S4: Optimized structures of diprotonated methylguanidine (mGuaH_2^{2+}) and 2-methylbutane (TMB) — models of Arg and Leu side chains. The distances between hydrogen atoms on different monomers, less than 2 Å, are shown explicitly. Also shown is the electrostatic potential mapped on the density isosurface drawn at 0.007 e/bohr^3. The range of the electrostatic potential in each figures is between -0.325 and 0.325 a.u.ESP. The density value and the range of ESP are the same as those used for the monoprotonated guanidine·TMB complex in the main text and Fig. S1.

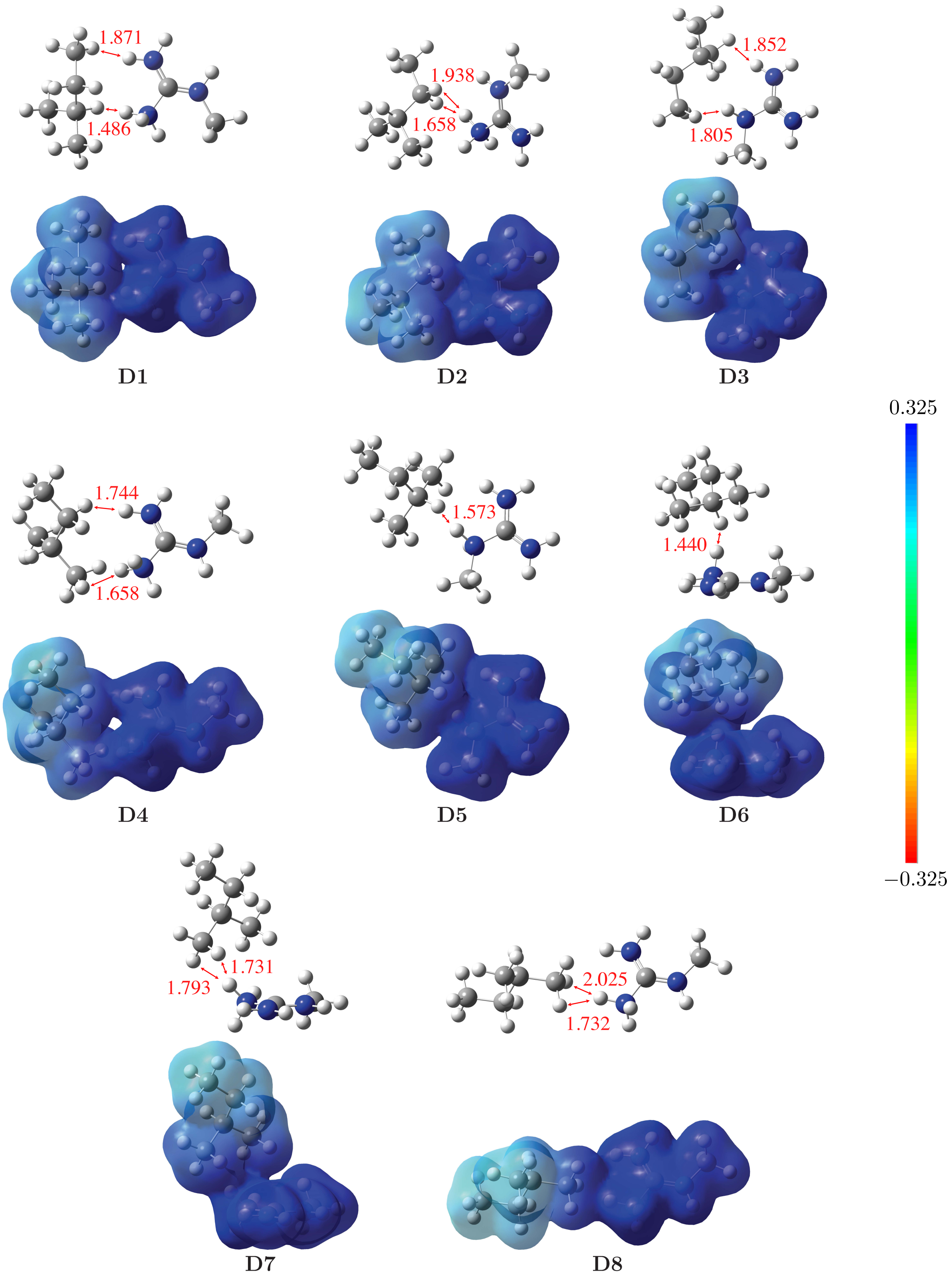

Figure S5: Examples of different types of structures with close approach between Arg and Leu side chains in the PDB: (a) PDB ID 3VLA, 7RWG, 2WFI; in the majority of the structures the methyl group of Leu interacts with one amino group of Arg; in different structures the methyl group approaches the guanidinium ion at various angles; (b) PDB ID 1GKM, 5D66, 7RWG; sandwich/stacking type of interaction; (c) PDB ID 3WH1, 2CNQ, 3DK9; aromatic rings in the vicinity of the Arg·Leu pair.

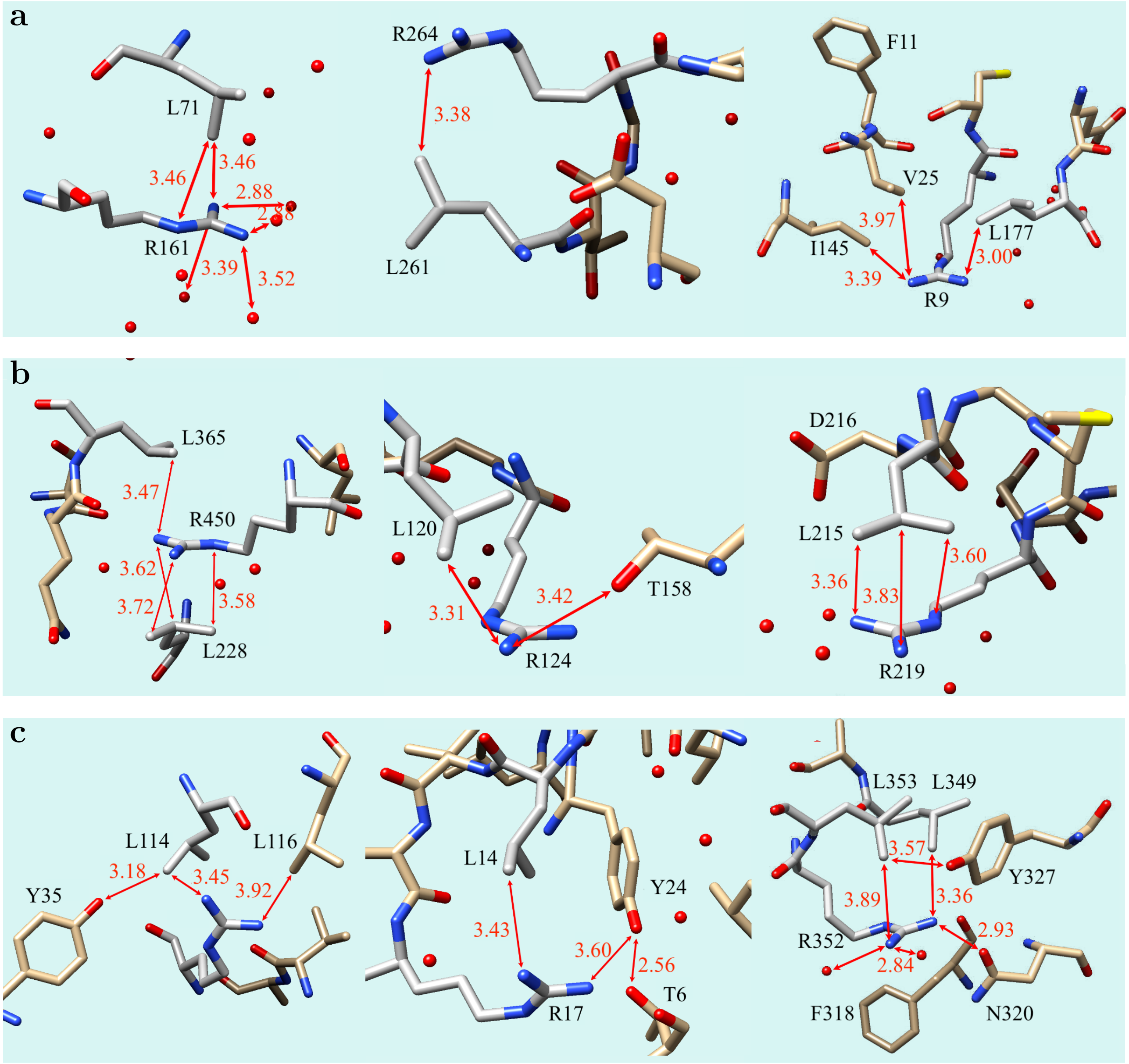

Figure S6: (a) The planar-like structure from the PDB ID 1MN8 file (Arg33·Leu13) . In this structure only one amino group of the Arg side chain interacts with one methyl of Leu. The structure in our calculations is a transition state between structures **P2** and **P3**. (b) The optimized transition state and the minima with their relative energies. In order to bring the second amino groups in close contact with TMB methyl, the fragments must undergo very large amplitude rotation. Such large amplitude motion should not be possible in a crowded environment of a protein, however, interactions with other groups of the protein, external chains, or water, can support such an arrangement. Note that the amino group close to Leu makes an even closer contact with Val36.

(**a**)

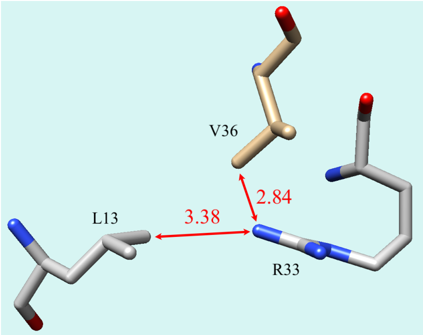

(**b**)

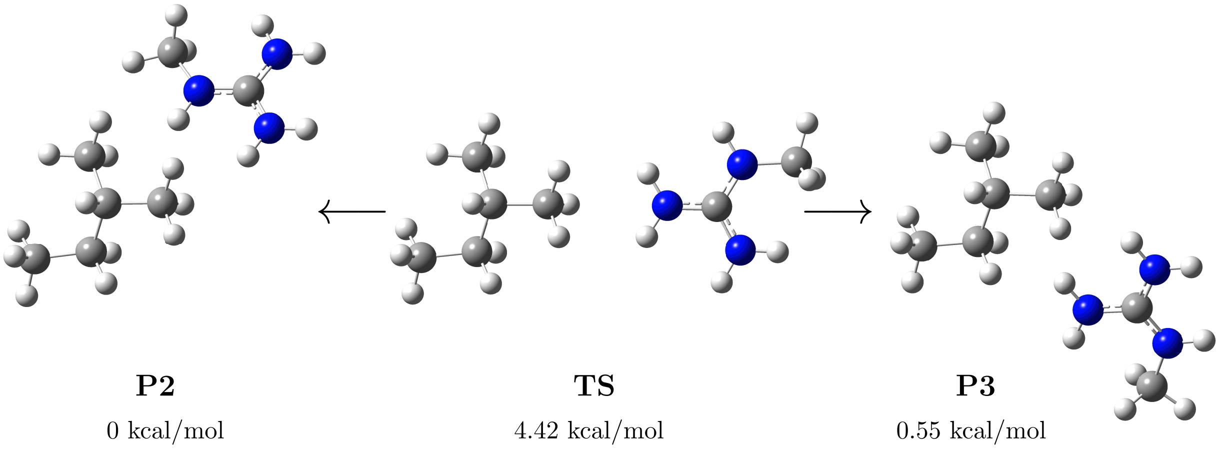

Table S1: Identified pairs with distances between any of the nitrogen atoms of Arg, and any of the carbon atoms of Leu shorter than 3.5 Å. Only the shortest distance for a given pair is included.

min. r(N−C) Å	PDB ID	Arg residue	Leu residue
2.92	5JIG	R86: NH2, chain A, alt.loc. B (0.50)	L63: CD1, chain A
3.00	2WFI	R9: NH1, chain A, alt loc B (0.40)	L177: CD1, chain A
3.09	5JIG	R76: NE, chain A	L63: CD2, chain A
3.18	3RQ9	R20: NH2, chain A, alt.loc.B (0.50)	L78: CD1 chain A
3.23	1MWQ	R21: NH1, chain B, alt. loc. A (0.60)	L25: CD1, chain B
3.24	1R6J	R197: NE, chain A, alt.loc. B (0.18)	L233: CD1, chain A
3.25	2XFR	R418: NH2, chain A, alt.loc. A (0.33)	L18: CD2, chain A
3.26	2XFR	R418: NH1, chain A, alt.loc. C (0.33)	L18: CD2, chain A
3.26	1MWQ	R21: NH2, chain B, alt. loc. B (0.40)	L18: CD2, chain B, alt loc. B (0.28)
3.28	5SBQ	R50: NH1, chain A	L64: CD1, chain A, alt.loc. A (0.70)
3.33	6UKF	R200: NH1, chain X	L222: CD2, chain X
3.36	3DK9	R352: NE, chain A	L349: CD1, chain A
3.36	7RWG	R219: NH1, chain A	L215: CD2, chain A, alt.loc. A (0.69)
3.38	1MN8	R33, NH2, chain D	L13, CD2, chain D
3.38	7RWG	R264: NH1, chain A	L261: cd1, chain A
3.39	5X9L	R76: NH2, chain A, alt.loc. A (0.50)	L75: CD2, chain A
3.39	6TGU	R229: NE chain A, alt.loc. B (0.30)	L293: CD1, chain A
3.40	1O7J	R140: NH2, chain A	L49: CD2, chain A
3.41	4GA2	R58: NH2, chain A, alt.loc. B (0.45)	L89: CD2, chain A
3.42	5NFM	R24: NH1, chain A	L26: CD2, chain A
3.43	7BBX	R330: NH2, chain A alt.loc. C (0.28)	L327: CD1, chain A
3.44	6EIO	R233: NH1, chain A	L52: CD1, chain A
3.44	3EO6	R58: NH1, chain A, alt.loc. B	L31: CD1, chain A
3.44	7B1S	R18: NE, chain C	L484: CD1, chain A
3.45	1GWE	R112: NH2, chain A	L190: CD1, chain A
3.45	3WH1	R134: NH1, chain A	L114: CD2, chain A
3.46	3VLA	R161: NH1, chain A	L71: CD2, chain A
3.46	7B1S	R18: NE, chain F	L484: CD1, chain D
3.46	1O7J	R140: NH2, chain D	L49: CD2, chain D
3.47	6TN1	R433: NH1, chain A, alt.loc. B (0.50)	L 372: CD2, chain A
3.47	1GKM	R450: NH2, chain B	L365: CD2, chain B
3.47	4U9H	R312: NE, chain L	L309: CD2, chain L
3.48	7P24	R185: NH2, chain A, alt.loc. A (0.60)	L 187: CD2, chain A
3.48	5GV8	R1163: NE, chain A, alt.loc. A (0.25)	L1159: CD1, chain A
3.48	5HB7	R196:NH2, chain A	L191:CD1, chain A
3.48	3X0I	R80: NH1, chain A, alt.loc. B (0.20)	L76: CD2, chain A
3.49	5E1N	R74: NH1, chain A, alt.loc. D (0.38)	L71: CD1, chain A, alt.loc. D (0.44)
3.49	2GKG	R46: NE, chain A, alt.loc. B (0.50)	L75: CD2, chain A
